# Leaching characteristics of metals from recycled concrete aggregates (RCA) and reclaimed asphalt pavements (RAP)

**DOI:** 10.1016/j.heliyon.2024.e30407

**Published:** 2024-04-30

**Authors:** Vu Quoc Hung, Ayomi Jayarathne, Chaminda Gallage, Les Dawes, Prasanna Egodawatta, Shiran Jayakody

**Affiliations:** a Faculty of Engineering, Queensland University of Technology (QUT), GPO Box 2434, Brisbane, 4001, Queensland, Australia; b Hydraulic Construction Faculty, National University of Civil Engineering, 55 Giai Phong Road, Hai Ba Trung District, Hanoi, Viet Nam; c Sustainable Engineered Construction Material Research Group, Faculty of Engineering, Queensland University of Technology (QUT), GPO Box 2434, Brisbane, 4001, Queensland, Australia

**Keywords:** Construction and demolition waste, Road construction, Metal leaching, Metal pollution, Recycled aggregates

## Abstract

Recycled concrete aggregates (RCA) and reclaimed asphalt pavements (RAP) are two construction waste products that are commonly used in the road construction industry. Besides many advantages, pollutants leaching from RCA and RAP are highlighted as the most concerning environmental issue. This study investigated metals leaching characteristics from RCA and RAP due to the variations in key influential factors of pH, dissolved organic carbon (DOC), compaction and liquid to solid ratio (L/S). The leaching tests for RCA and RAP were carried out separately and additionally, the standard leaching test was conducted as the benchmark for leaching investigations. Study outcomes revealed that the combined influences of factors are variable for RCA and RAP, while influences are also variable for individual metals. L/S ratios considerably affect the release of metals from RCA under saturated conditions, facilitating high metal concentrations in the leachate. On the other hand, acidic solutions are more favourable for leaching of metals from RAP. The influence of DOC in solution was minimal on the metal leachability. Interestingly, the increased degree of compaction with a higher density of materials presented the highest negative influence on metal leachability, suggesting that the metal leachability can significantly reduce, in particular when the RCA and RAP are used for the sub-base layers of road structure with a higher degree of compaction. However, the use of these recycled materials under field conditions should be further studied as there is an increasing concern of metal leaching from RCA and RAP with respect to recreational and drinking water thresholds.

## 1 Introduction

The demand for natural aggregates in the pavement industry is continuously increasing with the development of infrastructure. This demand escalates significantly when dealing with expansive soft subgrade conditions [1,2]. There are alternatives, such as employing geogrids in pavement structures to decrease the thickness of granular layers, thereby reducing the demand for natural aggregates [3,4]. However, recycled materials are the best alternative to replace natural aggregates, and recycling construction waste materials such as demolished concrete and reclaimed asphalt products is popular in the pavement industry. Such waste products require significant land space for disposal due to non-degradable and non-incinerable characteristics. Therefore, recycling is regarded as one of the sustainable waste management practices [[5], [6], [7]]. In the construction industry, these waste products are commonly recycled for road construction and low-grade concrete production where there is a high demand for crushed rock aggregates [8]. Recycled concrete aggregates (RCA) and reclaimed asphalt pavements (RAP) are the most widely used recycled materials as alternatives for quarry products. This is a viable option due to the limited availability and high costs of natural aggregates and both RCA and RAP having similar mechanical properties to natural aggregates [9,10]. RCA and RAP are primarily used for the construction of the base and sub-base layers of road pavements [[11], [12], [13], [14], [15], [16]]. For example, around 96 % of RCA was used as a sub-base of structural roads in Japan [17] and in the construction industry in Australia [18].

Besides the benefits, environmental issues related to the use of RCA and RAP, particularly the leaching of chemical pollutants, have been a critical concern [[19], [20], [21]]. The die-off of vegetation due to high-pH and high concentration of metals leaching from RCA are reported by Sakanakura et al. [5]. Furthermore, the leaching of organic compounds from RAP is also a potential consideration [22]. The leached chemical pollutants can potentially cause human health impacts via direct exposure or consumption when mixed with ground and surface water resources [23].

Leaching of metals from recycled materials is a complex process and can be influenced by a range of factors, which can be categorised as physical, chemical and external [24]. Fundamentally, these factors are not independent and exhibit an interactive influence. For example, percolation is one of the key physical processes that influence pollutant leaching while factors such as porosity and particle size of granular aggregates exert a significant influence on the percolation ability [25,26]. Diffusion, porosity and tortuosity significantly affect the movement of pollutants with leachate and are influenced also by compaction and particle size of the material [24,27]. In terms of chemical factors, dissolution, carbonation and acid/base buffering are three key factors influencing the pollutant leaching. López Meza et al. [28] highlighted that pollutant leaching is significantly affected by external factors such as the opportunity for contact with water [29]. In addition, metal leaching can increase rapidly with the presence of dissolved organic carbon (DOC) in percolating water [24]. Collectively, influences by these factors can be hypothesized to be governed by five major factors. These factors are amount of water content (denoted by liquid to solid ratio L/S), pH, DOC in seeping water, particle size and compaction [24,[30], [31], [32]]. These five factors were selected in this study as critical due to their practical relevance in geotechnical engineering in terms of road sub-base and backfill designs.

Even though a significant amount of research has been carried out to characterise the influence of separate factors on metal leaching from recycled materials, the studies relating to the combined effect of influential factors are limited. Most of the current investigations have only been focused on one or two influential factors. For examples, Komonweeraket et al. [33], Kosson et al. [32] and Chen et al. [34] investigated the effects of pH on pollutant leaching from construction materials, while López Meza et al. [29] investigated the influence of contact time and volume of liquid exposed to solid on leaching. Furthermore, leaching studies are limited to a selected number of metals and water quality parameters, disregarding potential leachability of trace metals that are common in RCA and RAP. Accordingly, the objectives of this study were to: (i) investigate the combined effects of influential factors on the leaching characteristics; and (ii) investigate the leaching characteristics of a wide range of metals from RCA and RAP. Leaching tests were conducted separately for RCA and RAP to estimate the effects of individual factors, such as L/S ratios, compaction, DOC content in percolating water, and pH. Additionally, a standard leaching test was employed as a benchmark for comparative analysis. The factors influencing metal leaching from both RCA and RAP were determined using advanced multivariate data analysis techniques, including Principal Component Analysis (PCA) and the Preference Ranking Organisation Method for Enrichment Evaluations (PROMETHEE). The results of the study led to the identification of critical leaching metals and the key factors influencing metal leaching. These findings are intended to improve the effective and safe utilization of RCA and RAP in road construction.

## 2 Material and methods

### 2.1 Test materials

**Fig. 1:**
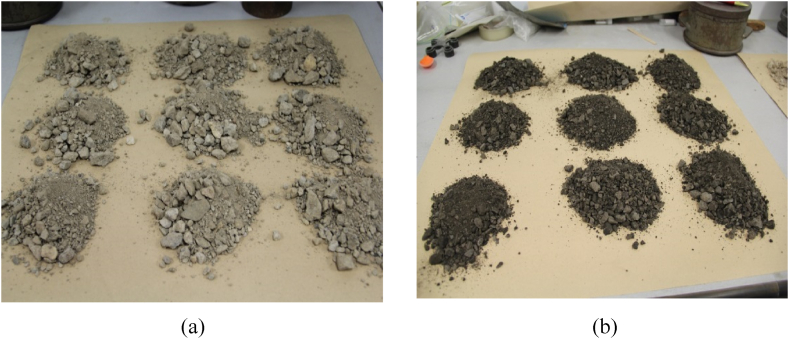
Test materials: (a) recycled concrete aggregates, (b) reclaimed asphalt pavements.

For this study, RCA and RAP samples were collected from the production plants operated by two leading producers of recycled materials for the civil construction industry in Queensland, Australia. Both production plants receive materials from state-wide locations and from variety of sources. Samples were collected from several stockpiles and mixed to create a homogeneous single sample. This was done to account for varied sources of crushed concrete and pavement milling. Mixed samples were then separated into portions for various testing. The samples used for the study are shown in Fig. 1(a) and (b). According to the standard compaction test [35], RCA achieved the maximum dry density of 1.75 g/cm^3^ at the optimum water content of 13.2 % and for RAP, they were 1.82 g/cm^3^ and 13.5 %, respectively. The specific gravity was measured as per Australian standards [36] and the values are 2.64 and 2.59, of RCA and RAP respectively.

### 2.2 Laboratory tests

Separated portions of samples were analysed for parameters listed in Table 1. Samples required for each test in Table 1 was prepared by sieving original RCA and RAP samples. All the tests listed in Table 1 were repeated three times from sample preparation for testing. Particle size distribution and mineralogy were analysed in order to determine the typical grain size and mineralogical phases in RCA and RAP. The leaching tests for RCA and RAP were carried out separately to understand the influence of individual factors including, L/S ratios, compaction, DOC content in percolating water and pH. In addition, the standard leaching test was conducted as recommended by NEN-734 [37] as the benchmark for leaching investigations. This was to compare the leaching characteristics of RCA and RAP samples used in this study with similar samples from other regions.

**Table 1:** Parameters and test methods.

Parameters	Test method	Comments
Particle size distribution	Sieving [29]	Sieve sizes from 63 mm to 0.063 mm were used.
Mineralogy	X-ray diffraction [30]	Samples were analysed using PANatycalX’Pert diffractometer.
Standard leaching	Leaching characteristic of building material [27]	L/S = 100 mL/g, pH = 4, particle size ≤2 mm and contact time = 48h
Leaching tests as a function of L/S	Compliance test for leaching of granular waste materials and sludge [31]	L/S values ranging from 0.1 mL/g to 100 mL/g were tested for particle size < 4 mm while maintaining other factors constant (pH = 7 and contact time = 48h)
Leaching tests as a function of pH	Leaching behaviour test with initial acid/base addition [32]	pH values ranging from 2 to 12 were tested while maintaining other factors constant (L/S = 10 mL/g, particle size <1 mm, contact time = 48h)
Leaching tests as a function of compaction	Compliance test for leaching [31] and compacted bulk density [33]	1.5 kg of dry material mixed with 13 % of water was compacted into each cylinder (70 mm diameter and 250 mm height) by applying different number of blows. The different compactions were achieved different number of blows (L/S = 10 mL/g, pH = 7, grain size <4 mm)
Leaching tests as a function of DOC/TOC	Compliance test for leaching [31] and method 5310C [34]	Tests were carried out with five different concentrations of DOC/TOC in leachate by maintaining other factors constant (L/S = 10 mL/g, pH = 7, particle size <4 mm and contact time = 48h)

A detailed description of laboratory test methods is provided in the Supplementary Information. In summary, specially designed small column apparatus as presented in Figure S1 in Supplementary Information was used to test at low L/S ratios (0.1, 0.5, and 1) and the effects of compaction on leaching characteristics. The concentrations of thirteen metals in leachate (Na, K, Al, Ca, Mg, Ni, Pb, Zn, Fe, Cd, Cr, Cu and V) were determined based on the USEPA method 200.8 [38] using inductively coupled plasma mass spectrometry (ICP-MS). Calibrations and internal standardisation were conducted to ensure the reliability of the metal analysis. The instrumental calibration was done by using ICP Quality Control Standard #3 (100 μg/mL in 5 % HNO_3_) provided by AccuStandard®. Five calibration standards were prepared including 0.5 ppb, 5 ppb, 50 ppb, 500 ppb and 5000 ppb. Deionised water with the same acid matrix with calibration standard was used as the calibration blank. Internal standards including Beryllium (Be), Indium (In), Bismuth (Bi) and Rhodium (Rh) with 1000 μg/mL in 1 % HNO_3_ were also prepared in accordance with the USEPA method 200.8 [38].

### 2.3 Data analysis

The factors influencing metals leaching from both RCA and RAP were identified using multivariate data analysis techniques of principal component analysis (PCA) and the Preference Ranking Organisation Method for Enrichment Evaluations (PROMETHEE). PCA is a data reduction technique that reduces the dimensions of data matrix. This technique creates new “inter-correlated variables” transforming the large set of original variables into the new orthogonal variables which are called ‘principal components’ (PCs) [39,40]. The first PC has the highest variance in the data matrix and presents the maximum information of variables, whilst other PCs account for decreasing order of variance. Prior to PCA, the data matrix was pre-treated by outlier detection and standardization to prevent different scale effects of variables. Further details of the PCA method can be found elsewhere [41].

PROMETHEE is a non-parametric statistical method that ranks the preference of one object to another for each variable in the data matrix [42,43]. The ranking is based on the preference and weighting of corresponding variables that have been pre-selected by the decision maker [41]. Further, the selected variables can be maximised or minimised depending on their influence. Then, a set of net ranking outflow values in term of φ, are calculated for individual objects. A detailed description of the PROMETHEE analysis can be found in Ayoko et al. [44].

## 3 Results and discussion

### 3.1 Characterisation of RCA and RAP

**Fig. 2:**
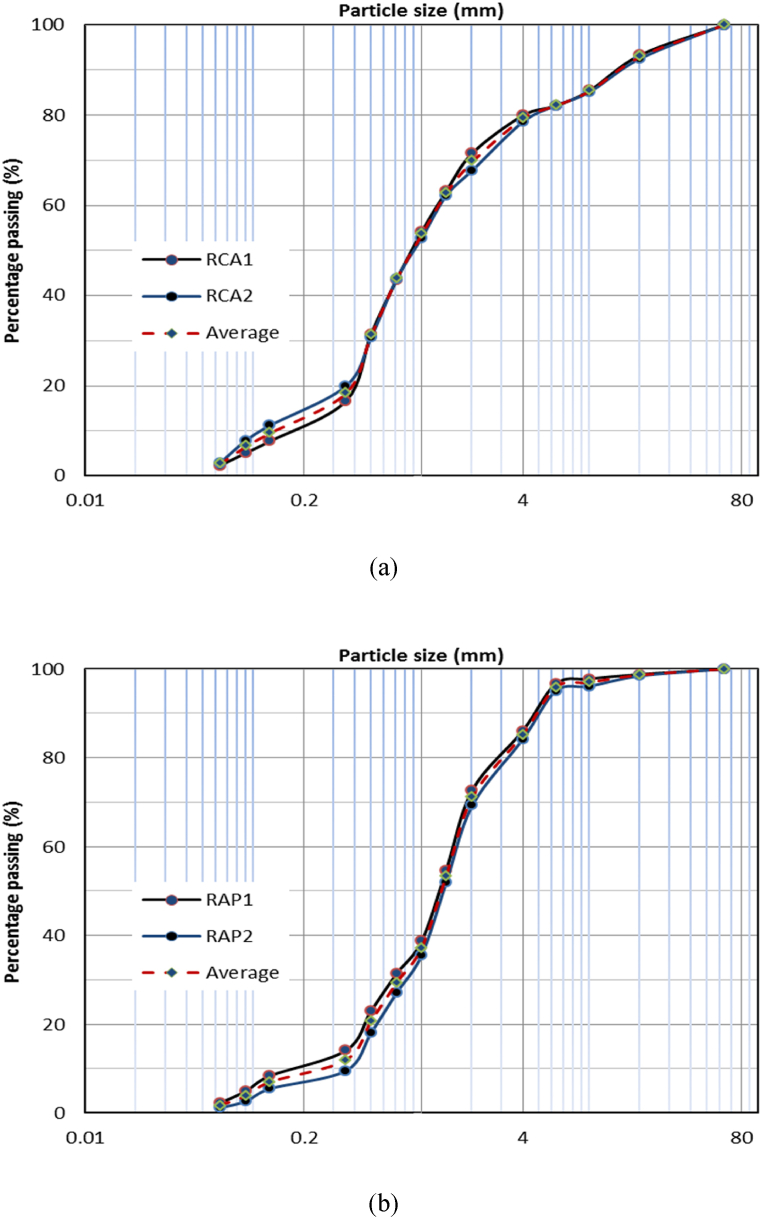
Cumulative particle size distribution of (a) recycled concrete aggregates (RCA) and, (b) reclaimed asphalt pavements (RAP).

The differences in particle sizes of materials can directly affect the leaching regime due to the changes in the surface area of aggregates contacting liquid. As pointed out by. Galvín et al. [45], the concentrations of metals leaching from recycled construction materials with finer aggregates is higher than those with coarser aggregates. The particle size distributions of two samples of each RCA and RAP are given in Fig. 2 (a) and (b) respectively. As evident from Fig. 2 (a) and (b), particle sizes less than 4 mm accounted for about 80 % of the material, implying a higher capacity for metal leaching from RCA and RAP.

**Fig. 3:**
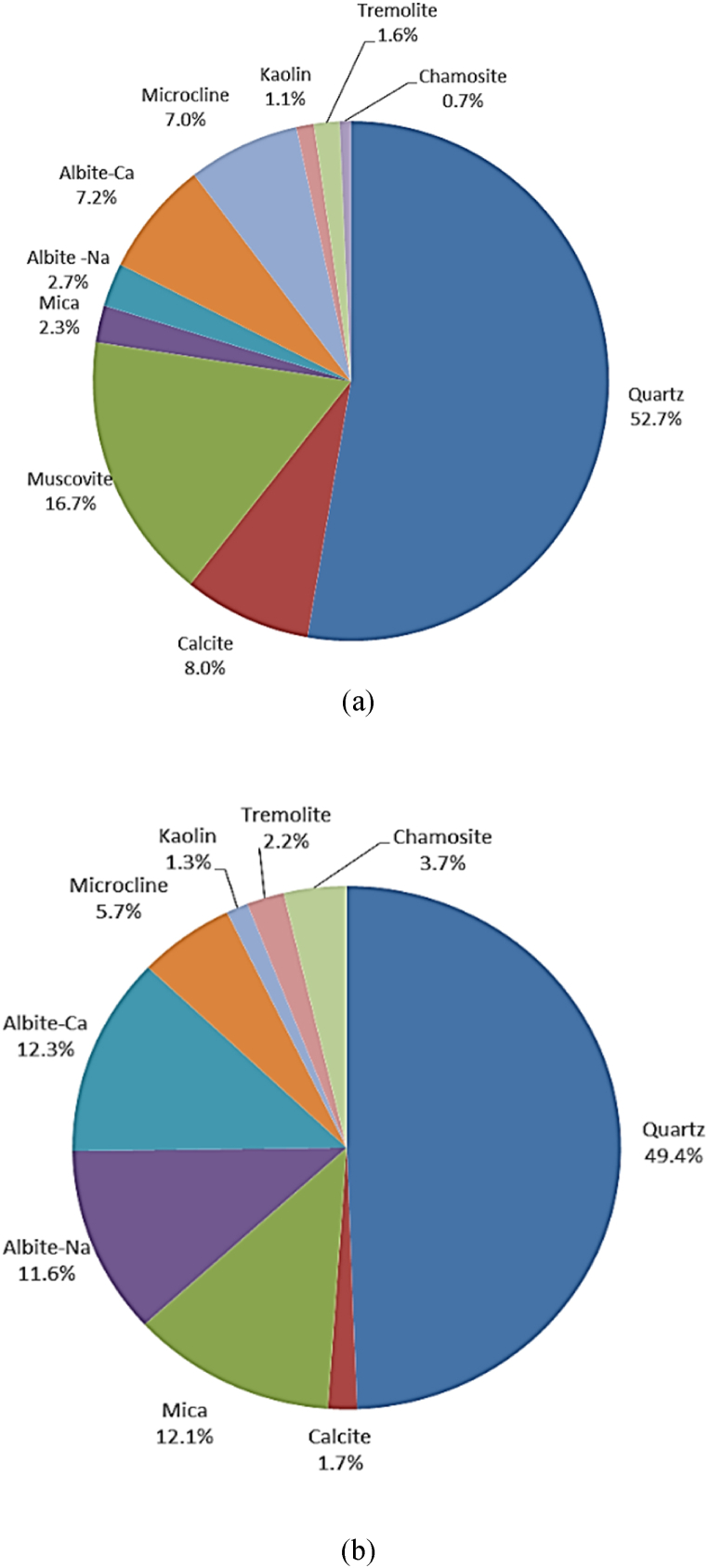
Mineralogical content of (a) recycled concrete aggregates (RCA); (b) reclaimed asphalt pavements (RAP).

The proportions of crystalline minerals found in RCA and RAP are shown in Fig. 3 (a) and (b). Quartz predominated in mineralogical composition, which can be attributed to the sand content that was derived from the establishment of original material [46]. About 27 % and 21.3 % of the total mineralogical composition of RCA and RAP, respectively, came from the mineral group that has an origin from sedimentary rocks, stones and limestone including Calcite, Muscovite, Mica, Tremolite and Chamosite. Another mineral group derived from brick and ceramic is the alkali-feldspars (Albite-Na, Albite-Ca and Microcline) that consisted of about 17 % in RCA and 30 % in RAP. The mineralogical composition exerts a direct influence on adsorption/desorption of metals to particles and thereby can control the metal solubility. Mineralogical composition presented in Fig. 3 excludes the amorphous content present in both RCA and RAP samples.

### 3.2 Variability in metal leaching with respect to threshold concentrations

**Fig. 4:**
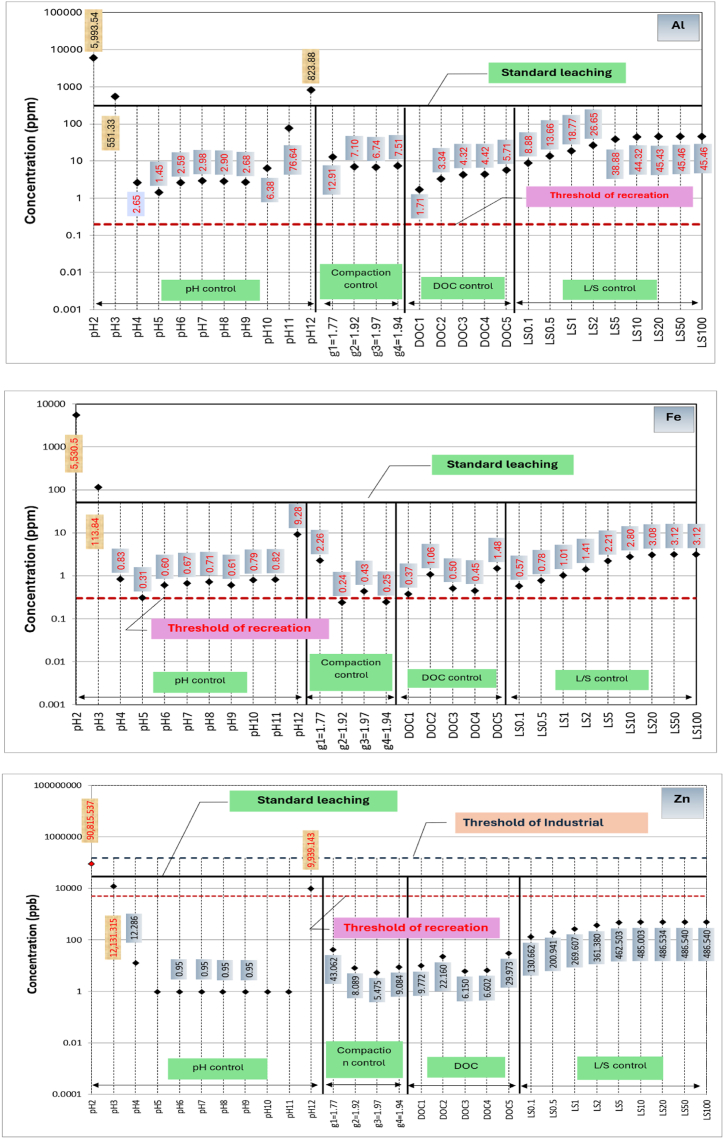

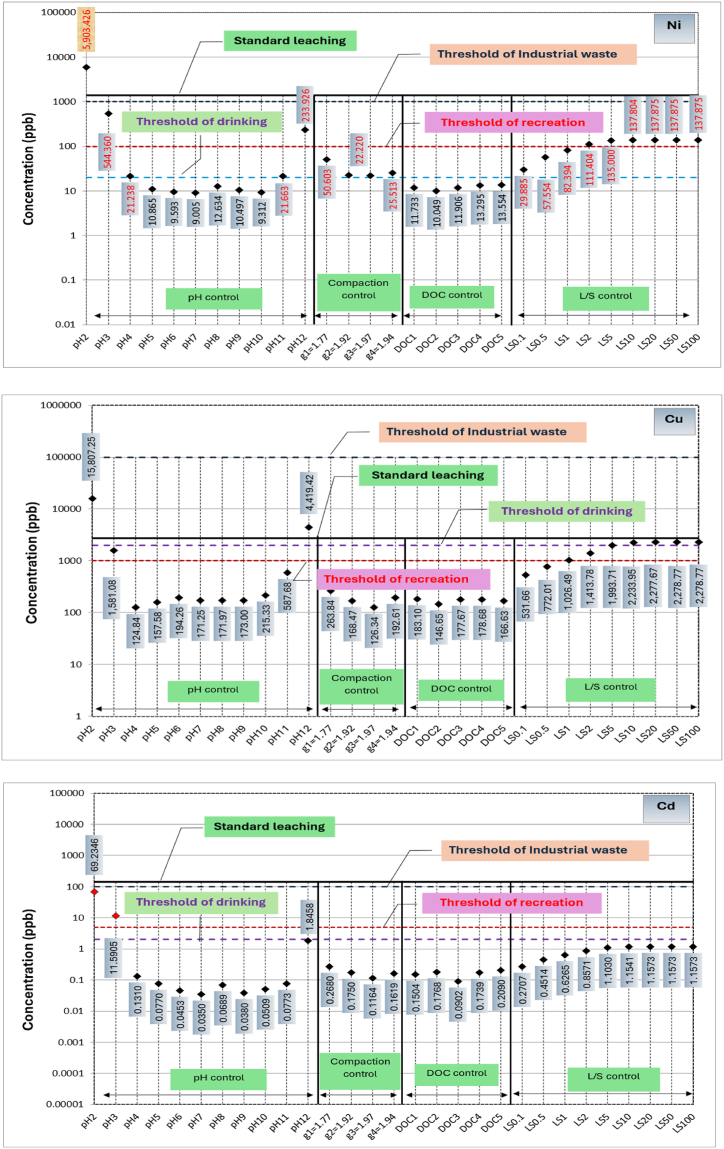

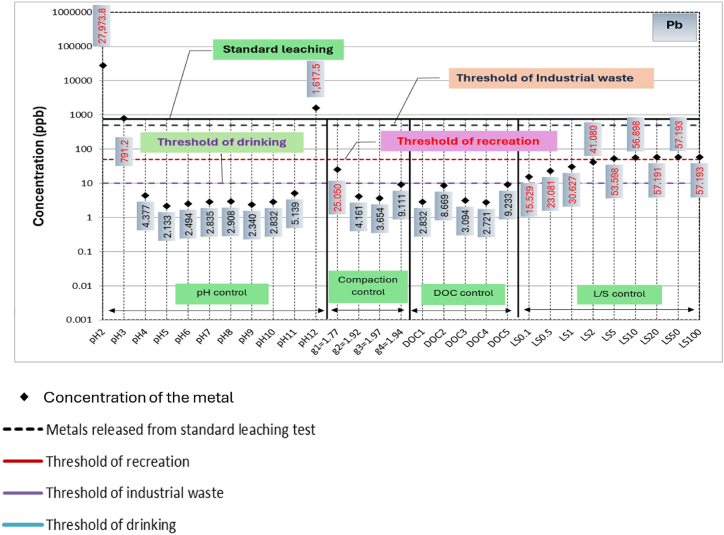
Concentrations of metals leaching from recycled concrete aggregates (RCA) in comparison to standard leaching test and water quality thresholds.

**Fig. 5:**
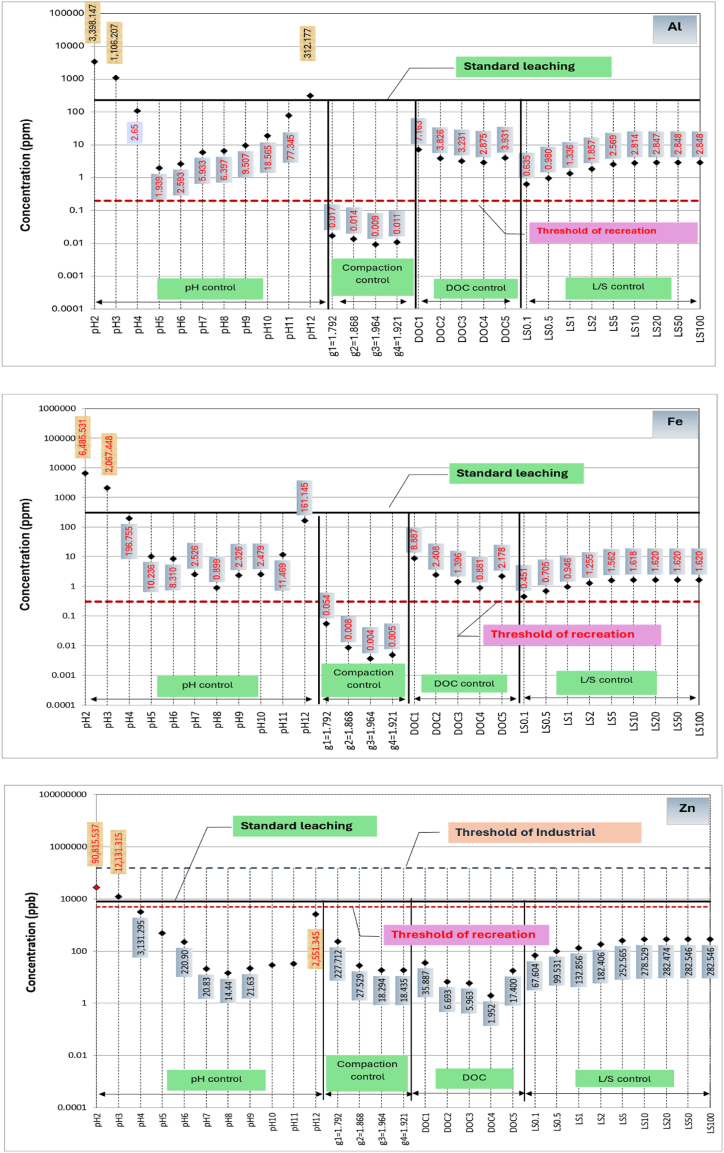

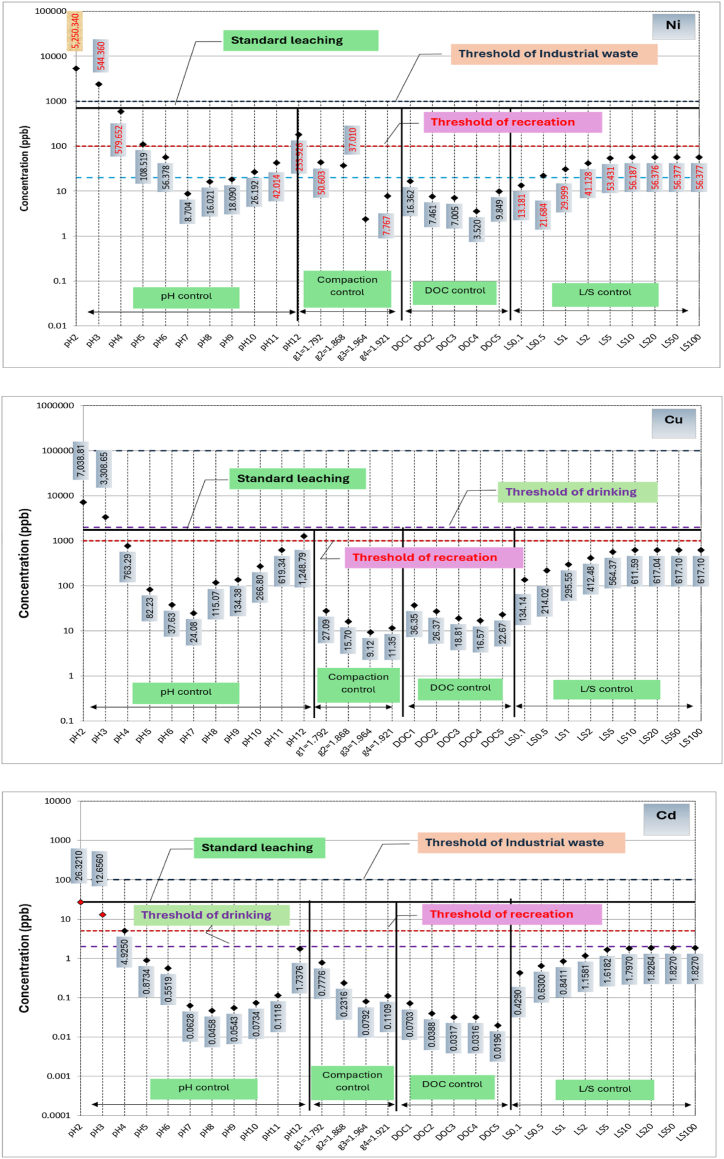

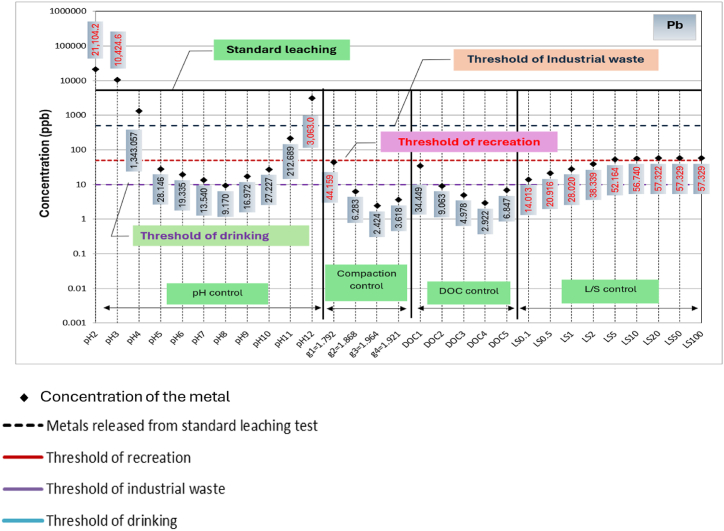
Concentrations of metals leaching from reclaimed asphalt pavements (RAP) in comparison to standard leaching test and water quality thresholds.

A comparison among metals leached from RCA and RAP due to variation in the primary influential factors, respective threshold concentrations for drinking water monitoring, recreational water monitoring and industrial waste effluent monitoring, and metals leached during standard leaching tests are shown in Fig. 4, Fig. 5, respectively. Based on the available thresholds, only seven metals, including Al, Fe, Zn, Ni, Cu, Cd and Pb are discussed. The threshold concentrations for water quality and public health risk are specified based on a range of guidelines [47]. Further, thresholds for leached liquid from solid wastes into the environment is primarily governed by the guideline relating to industrial waste management [48].

As shown in Fig. 4, Zn and Cd concentrations leached out from RCA due to all major factors are lower than the respective industrial waste disposal, recreational and drinking water thresholds except during extreme pH values. However, Al and Fe leaching from RCA can be considered as critical for water sources used for recreational purposes as their concentrations are well above the threshold for most of the conditions. Even though low level exposure to Al and Fe does not cause adverse effects on human, chronic exposure to Al may cause nerve damage, kidney damage and allergies [49,50], and genetic and metabolic diseases by Fe [51].

On the other hand, water insoluble Fe (3+) can cause undesirable tastes and colour, preventing usage of water for drinking and recreation purposes. As evident from Fig. 4, Ni, Cu and Pb concentrations leached out are larger than the respective drinking water quality thresholds and lie close to recreational water quality thresholds. A high level of Cu usually leaves a metallic or unpleasant bitter taste in the drinking water [52], whilst Pb exposure for young children via ingestion can impair mental function [49]. Furthermore, chronic exposure to Ni via drinking water can cause carcinogenic effects [53]. Thus, it can be postulated that Ni, Cu and Pb leaching from RCA can potentially cause human health impacts when RCA is used close to drinking or recreational water resources.

In the case of RAP, as evident from Fig. 5, the leaching of Cd, Ni, Cu and Pb lies close to the threshold concentrations for recreational water use and exceeds under extreme pH conditions (pH 2, pH3 and pH 12). In terms of drinking water thresholds, Cd, Ni, Cu and Pb concentrations in leachate are significantly higher for most of the conditions. These facts suggest that Cd Ni, Cu and Pb poisoning is a possibility when RAP is used in close proximity to water sources that use for drinking and recreational purposes. The threshold concentrations for recreational water use in terms of Al and Fe, as well as their concentrations from standard leaching, are enclosed in their concentration circles created by all influential factors, apart from compaction.

### 3.3 Factors influencing the leaching mechanism of RCA and RAP

An overall assessment of the combined effect of all influential factors of metal leaching from RCA and RAP enables the replication of more realistic field conditions. Accordingly, PCA and PROMETHEE analyses were undertaken to provide a comprehensive evaluation and ranking of the influential factors. The assessment was performed for major metals (Na, K, Al, Ca and Mg) and trace metals (Ni, Pb, Zn, Fe, Cd, Cr, Cu and V) separately for selected recycled materials. Data for pH above 10 and less than 5 were disregarded for this analysis to prevent obscured outcomes. The data matrix consisted of major/trace metals concentrations and 54 objects representing changes to (ranging from 5 to 10), DOC, L/S ratio and compaction experiments.

**Fig. 6:**
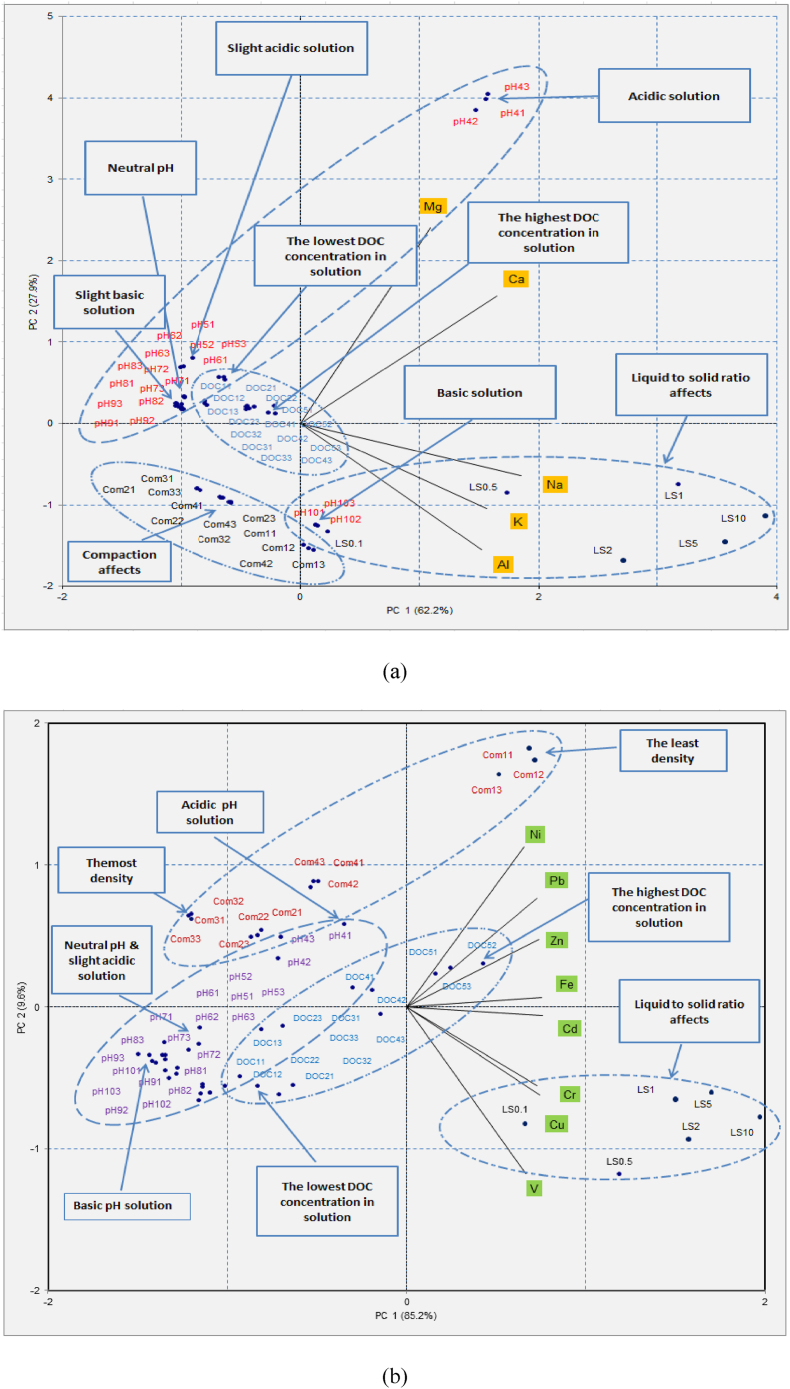
PCA biplots representing the factors influencing the leaching of (a) major metals and (b) trace metals from recycled concrete aggregates (RCA).

As evident from the PCA biplot for major elements leached from RCA (see Fig. 6(a)), changes in L/S ratio provide the highest variance in the direction where Na, Al and K are pointed. This indicates that the leaching of these metals is highly influenced by the L/S ratio. It is also noted that alkaline water can increase the leaching of these metals. Two metal species, Mg and Ca, are pointed towards the general direction where the objects indicating acidic pH changes, while the influence of other factors can be considered minimal. In terms of trace metals (see Fig. 6(b)), Ni, Pb and Zn are influenced by the varying compaction and DOC, and for a lesser extent by L/S ratio. The other group, which includes Cr, Cu and V shows a weak negative relationship with changes to compaction, pH and DOC, but a strong positive relationship with changes to L/S ratio. This implies that the leaching of Cr, Cu and V from RCA is strongly influenced by the changes to L/S ratio, whilst the degree of influence by pH, DOC and compaction is less. These observations indicate the differences in leaching behaviour and variations in the degree of influence by different factors on different metal elements.

**Fig. 7:**
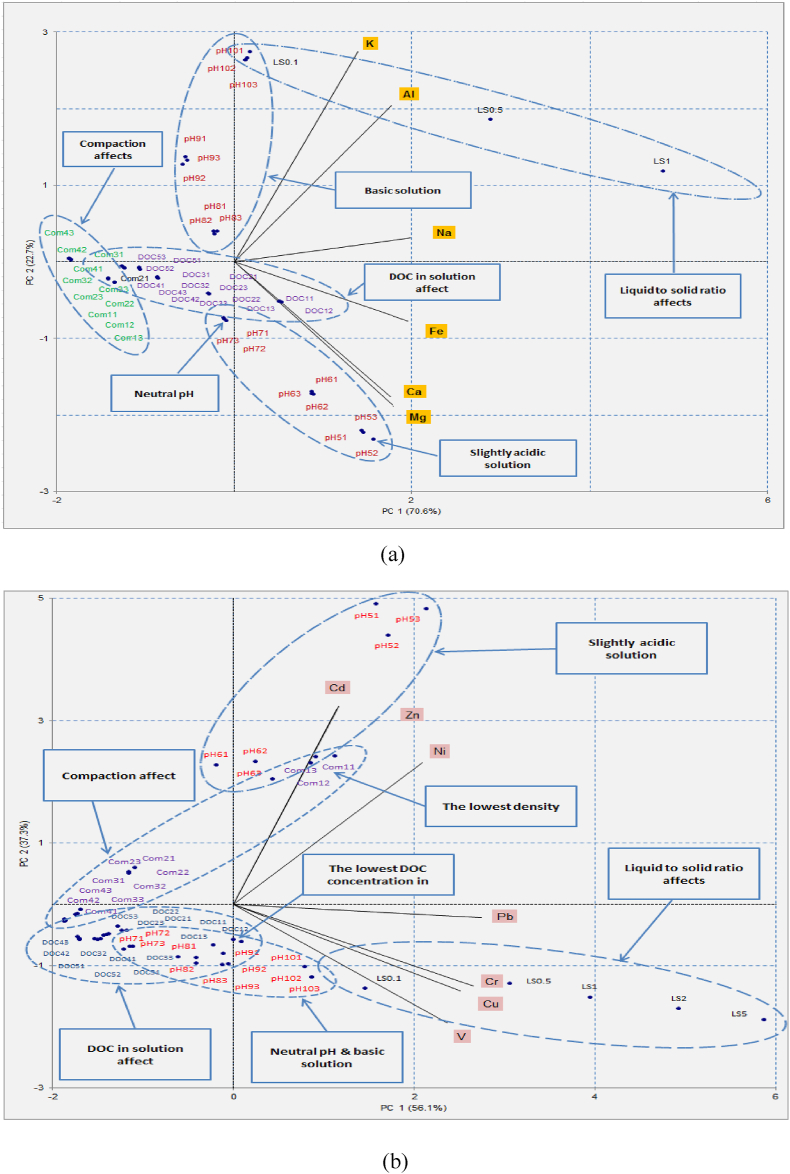
PCA biplot representing the factors influencing the leaching of (a) major metals and (b) trace metals from reclaimed asphalt pavements (RAP).

The PCA biplots representing the factors influencing metal leaching from RAP are shown in Fig. 7. As shown in Fig. 7(a), major metals of K, Na and Al are highly influenced by L/S ratio, and to a lesser extent, by alkaline solutions. Leaching of another major metal group, which contains Fe, Ca and Mg influences by DOC in solution and acidic pH conditions. As evident from Fig. 7(b)–a group of trace metals including Cd, Zn and Ni are correlated to objects with mild acidic solutions and lower compaction. On the other hand, Pb, Cr, Cu and V are strongly affected by low L/S ratios (L/S from 0.1 to 1). Leaching of these metals are also influenced by compaction, alkaline pH values and DOC to a lesser extent.

The analyses for both major and trace metals leached from RCA and RAP indicate the differences in leaching behaviour and variations in the degree of influence by different factors for different metal elements. Therefore, it is essential to have an assessment of the overall influence of factors on metal leaching. For this, the PROMETHEE ranking was undertaken for the same data matrix used for PCA analysis. In PROMETHEE analysis, equal weighting was used for all the variables with the “V-shaped” preference function. Accordingly, the net out-ranking flow values (φ) were evaluated to understand the degree of influence exerted by different factors (see Table 2).

**Table 2:** An overall ranking of influential factors on metal leaching from recycled concrete aggregates (RCA) and reclaimed asphalt pavements (RAP).

Ranking	For RCA		For RAP		Comments
	Factors	Net outranking flow (φ)	Factors	Net outranking flow (φ)	
1	L/S = 10	0.6236	pH4	0.6845	High positive influence on leaching High negative influence on leaching
2	L/S = 5	0.5848	L/S = 10	0.3554	
3	L/S = 2	0.442	L/S = 5	0.3202	
4	L/S = 1	0.2357	pH5	0.2749	
5	Com1	0.066	L/S = 2	0.1901	
6	L/S = 0.5	0.0551	pH6	0.1114	
7	DOC5	0.0363	pH10	0.0711	
8	pH4	0.0062	L/S = 1	0.0693	
9	DOC1	−0.0445	DOC1	0.0675	
10	DOC4	−0.0522	pH9	−0.0191	
11	pH10	−0.0553	L/S = 0.5	−0.0419	
12	DOC3	−0.0612	DOC2	−0.0575	
13	DOC2	−0.0927	pH8	−0.0582	
14	L/S = 0.1	−0.1127	Com1	−0.0689	
15	Com4	−0.161	pH7	−0.0924	
16	Com2	−0.1806	DOC3	−0.1815	
17	pH6	−0.1909	L/S = 0.1	−0.1861	
18	pH9	−0.2017	DOC5	−0.1918	
19	pH8	−0.2098	Com2	−0.2578	
20	pH5	−0.2182	DOC4	−0.2691	
21	pH7	−0.2201	Com4	−0.3541	
22	Com3	−0.2489	Com3	−0.3663	

As observed in Table 2, L/S ratios ≥1 condition (saturated conditions) has the most positive influence on metal leaching from RCA, while high pH values (alkalinity) and high compaction exert the most negative influence. This implies that metals are likely to be leached from recycled waste materials under high L/S ratios, whilst highly compacted materials have provided limited opportunities for water contact and hence potential leaching of metals. Based on this, it can be suggested that the metal leachability from RCA is significantly influenced by L/S ratio, compaction and pH variations. Having DOC concentrations close together in the mid-range of ranking suggests that its influence on metal leaching is minimal. Acidic solutions (pH 4 to 6) and high L/S ratios present the highest positive influence on metal leaching from RAP, while a high degree of compaction presents the highest negative influence. High DOC concentrations also have a minor negative influence on metal leaching from RAP.

## 4 Conclusion

Leachate from RCA and RAP add additional metal sources, increasing the risks to environmental and human health due to long-term exposure and bioaccumulation, despite potential dilution in larger water bodies. This study analysed the metal leaching characteristics from RCA and RAP due to the variations in key influential factors of pH, DOC, compaction and L/S ratio and the significant conclusions drawn from the analysis are outlined below.

• The study identified critical levels of Al and Fe leaching from RCA, exceeding thresholds set for recreational water use. Additionally, Ni, Cu, and Pb leaching from both RCA and RAP approached threshold concentrations, suggesting potential harm to human health and the environment near water resources.
• High L/S ratios and alkaline conditions exert the greatest influence on the leaching of metals such as Na, Al, and K from RCA. Conversely, metals like Mg and Ca are affected by acidic pH ranges, while Ni, Pb, Zn, Fe, and Cd are influenced by variations in compaction and DOC content. In the case of RCA, changes in L/S ratio strongly impact the leaching of Cr, Cu, and V. Similarly, in RAP, leaching of metals such as Na, Al, and K is primarily influenced by high L/S ratios.
• According to the PROMETHEE ranking, saturated conditions with high L/S ratios show the greatest positive influence on metal leaching from RCA. For RAP, acidic solutions (pH 4 to 6) and high L/S ratios demonstrate the strongest positive impact on metal leaching. The high compaction of both RCA and RAP significantly hampers leaching due to the limited water contact in densely packed materials.
• The outcomes offer valuable insights into the use of RCA and RAP in field applications. An important measure identified is the necessity to minimize water percolation and groundwater seepage through RCA and RAP layers, given the significant impact of L/S ratio on metal leaching. Compaction during application is vital, as it can mitigate leaching effects and reduce material porosity, thereby minimizing water infiltration. Moreover, caution should be exercised when using RCA and RAP near sensitive water environments and acidic soil conditions, as this may lead to adverse effects.

**Table:** Data availability statement

Question	Response
Data Availability Sharing research data helps other researchers evaluate your findings, build on your work and to increase trust in your article. We encourage all our authors to make as much of their data publicly available as reasonably possible. Please note that your response to the following questions regarding the public data availability and the reasons for potentially not making data available will be available alongside your article upon publication. Has data associated with your study been deposited into a publicly available repository?	No
Please select why. Please note that this statement will be available alongside your article upon publication. as follow-up to “Data Availability Sharing research data helps other researchers evaluate your findings, build on your work and to increase trust in your article. We encourage all our authors to make as much of their data publicly available as reasonably possible. Please note that your response to the following questions regarding the public data availability and the reasons for potentially not making data available will be available alongside your article upon publication. Has data associated with your study been deposited into a publicly available repository?	Data will be made available on request

## CRediT authorship contribution statement

**Vu Quoc Hung:** Writing – original draft, Methodology, Investigation, Formal analysis, Data curation, Conceptualization. **Ayomi Jayarathne:** Writing – original draft, Visualization, Validation, Software, Methodology, Investigation. **Chaminda Gallage:** Writing – review & editing, Validation, Supervision. **Les Dawes:** Writing – review & editing, Supervision. **Prasanna Egodawatta:** Writing – review & editing, Validation, Supervision, Resources, Project administration, Funding acquisition. **Shiran Jayakody:** Writing – review & editing, Validation.

## Declaration of competing interest

The authors declare that they have no known competing financial interests or personal relationships that could have appeared to influence the work reported in this paper.
